# Brazilian Reference Percentiles for Bioimpedance Phase Angle of Healthy Individuals

**DOI:** 10.3389/fnut.2022.912840

**Published:** 2022-07-06

**Authors:** Rita Mattiello, Eduardo Mundstock, Patrícia Klarmann Ziegelmann

**Affiliations:** ^1^Programa de Pós-graduação em Epidemiologia, Universidade Federal do Rio Grande do Sul, UFRGS, Porto Alegre, Brazil; ^2^Pontifícia Universidade Católica do Rio Grande do Sul, PUCRS, Porto Alegre, Brazil; ^3^Secretaria da Educação, Esporte e Lazer de Canela, Canela, Brazil

**Keywords:** bioimpedance (BIA), phase angle (PA), reference values, percentiles, determinants

## Abstract

**Objectives:**

The present study was designed to estimate phase angle percentile curves for a broad age range of healthy individuals.

**Methods:**

This is a cross-sectional study of healthy Brazilian individuals aged five to 80. InBodyS10 was used to assess phase angle. Reference curves were stratified by sex and estimated using Generalized additive models for location, scale, and shape as a continuous function of age. The phase angle determinants analyzed were physical activity, age, BMI, and SES variables.

**Results:**

Data were analyzed from 2,146 individuals, 1,189 (55.2%) of whom were female. In both sexes, the phase angles showed a similar pattern (an increasing trend from childhood to the teenage phase, followed by stabilization during adult ages and a decrease in old adults). In female, the relationship between phase angle and age were associated with BMI and family income. In the male, the relationship between phase angle and age were associated with skin color and family income.

**Conclusions:**

To the best of our knowledge, it is the first attempt to apply the GAMLSS technique to estimate phase angle percentiles in a healthy population covering most of the life cycle. We also showed that there are different phase angle determinants according to sex.

## Introduction

Phase angle (PA) from bioimpedance is measured by the potential difference of a low voltage alternating electric current introduced into the body. It is dependent on the resistive behavior and the capacitive effect on the cell membrane and other interfaces (1). PA has been proposed to indicate cellular health, where higher values reflect higher cellularity, cell membrane integrity, and better cell function (1). For this reason, it has been used as a health status tool and an important predictor of disease severity and survival in different medical conditions (2–6). However, the cut-offs used in the literature are not necessarily transferable to other populations and might thus not be applicable in the general clinical setting. This is because the cut-offs are generated primarily using the median or lowest quantile from a specific population without considering the determinants of phase angle. Phase angle reference values are still scarce, especially PA percentile curves (2).

According to one recent meta-analysis involving more than 250,000 healthy subjects, age, sex, and BMI seem to be main independent determinants of phase angle (7). Another meta-analysis showed that physical activity also influences PA values, especially in individuals with chronic diseases, indicating that it should be considered as an associated variable (8).

Therefore, the present study was designed to estimate phase angle percentile curves for a broad age range of healthy individuals stratified by sex and understand the relationship between phase angle and physical activity, age, BMI, skin color, and family income.

## Materials and Methods

### Study Design

This was a cross-sectional study that followed the STROBE statement guidelines for reporting observational studies (9).

### Setting and Participants

Healthy community-dwelling individuals aged five to 80 years old, of both sexes, were invited to participate in the study. The exclusion criteria were contraindications against bioimpedance measurements, such as diseases affecting the skin’s electrical resistance, pregnancy, persons with an implanted pacemaker or cardioverter-defibrillator, and amputated persons using a prosthesis/orthosis. Participants were considered healthy if they had not been diagnosed with any chronic disease or were not on continuous medication. Data were collected from December 2015 to April 2019 in public and private schools, companies, and at events in cities in southern Brazil. Recruitment occurred through word of mouth.

### Data Measurements

Sociodemographic variables were obtained through structured interviews. These included age (years), sex (male or female), self-reported skin color (categorized into white, black, or others—brown, Asian, and indigenous were grouped together to homogenize the size of the categories), and location of residence (rural or urban), defined according to the IBGE Brazilian demographic census (10). Income was categorized into low and high (according to whether the families earned more or less than the median income, calculated separately for the samples of men and women).

To assess the level of physical activity, the participants answered different validated questionnaires, according to their age. The children up to 10 years of age answered the Physical Activity Checklist (11), and participants over 10 years of age answered the short version of the International Questionnaire on Physical Activity (IPAQ) (12, 13). After that, the participants were classified as active or inactive according to their physical activity level. The cut-off points to be considered active was 300 min of moderate to vigorous physical activity (MVPA) per week for children and adolescents. For the adults (18 years and older), it was 150 min of MVPA, or 75 min of vigorous physical activity (VPA) per week. These cut-off points are the same ones suggested by the World Health Organization (14).

Body mass was measured with the participants in a standing position, wearing the least possible amount of clothing and no shoes, using a calibrated digital scale (Charder MS6121). Height was measured with the participants standing barefoot with their feet parallel and heels together, arms along their body, and head in the Frankfurt plane, using a Sanny compact stadiometer and a tape measure to the nearest 0.1 cm (American Medical do Brazil Ltda, São Bernardo do Campo, Brazil). Body mass index was classified as underweight, normal weight, pre-obesity, and obesity according to the WHO BMI classification for children, adults, and the elderly (15).

Bioimpedance Multi-frequency InBodyS10 (Ottoboni, Rio de Janeiro, RJ, Brazil) was used to assess phase angle. The InBodyS10 showed excellent agreements with DEXA regarding to whole body lean mass, fat mass and percentage body fat (16). The applied current was 100 μA (1 kHz) and 500 μA and frequency was 50 kHz. The hand electrodes were attached to each thumb and middle finger, while the foot electrodes were positioned between the ankle bone and the heel, covering as much area as possible. The BIA was performed with the participants on a non-conductive surface in the standing position, with their legs apart and arms held away from their body and wearing the least amount of clothing possible and no metal jewelry. The standard guidelines were followed to instruct regarding fasting state of the subjects before the BIA (17). All measurements were performed by one of the four experienced researchers according to the manufacturer’s instructions using a standardized technique. All the participants completed three evaluations, and the average of the three values was considered as their result.

### Statistical Analysis

The data were expressed as mean (SD) or median and interquartile range (IQR, 25th–75th percentiles) for the continuous variables and absolute and relative frequencies for the categorical variables.

Generalized additive models for location, scale, and shape (GAMLSS) were used to estimate age-related phase angles and determine phase angle predictors. These models are more flexible than linear or generalized linear models. They let the data determine the relationship between the predictor and the covariables rather than enforcing a linear (or polynomial) relationship. It is also possible to use smoothing techniques and allow the covariables to model variability and shape besides the median values (18, 19).

First, the LMS R function was used to estimate the power value, possible transformation of age and select the distribution family (among BCCGo, BCPEo, and BCT families). To identify the models’ optimum number of effective degrees of freedom (edf), the automated “pb” function was implemented. Models were also tested with and without age transformation, different degrees of freedom, and cubic smoothing. The models were compared using GAIC (generalized Akaike information criterion) (19, 20).

GAMLSS models were also used to explore phase angle determinants. Physical activity, BMI, skin color, and family income were analyzed one by one in a bivariable model with age to test any possible interaction between age and the covariables. The covariables and interactions, significant at 5%, were maintained in the multivariable model.

All analyses were performed using the R software, version 3.2.3, with the “gamlss” package, version 5.1-5 (21).

The present study was part of an umbrella project and was conducted according to the Declaration of Helsinki (22). This project received the approval of the Research Ethics Committee of the Pontifical Catholic University of Rio Grande do Sul (permission 2.187.802). For adult participants, the Informed Consent Term (ICT) was signed. In the case of underage participants, the consent term was obtained, and ICT signature was acquired from their parents or guardians.

## Results

A total of 2,122 participants aged five up to 80 years old were evaluated. The majority were females (*n* = 1,171, 55.2%); were aged between five and 12 years (*n* = 901, 42.4%); were underweight/normal weight (*n* = 979, 46.1%); self-declared their skin color as white (*n* = 724, 44.9%); were living in an urban area (*n* = 1,304, 61.5%); were inactive according to their physical activity level (*n* = 1,078, 67.4%); and were classified as low income (*n* = 846, 53.2%) (Table 1).

**TABLE 1:** Characteristics of participants.

Characteristics	Total sample	Male	Female
*n* (%)	2,122 (100.0)	951 (44.8)	1,171 (55.2)
Age (years), n (%)	2,122 (100.0)	951 (44.8)	1,171 (55.2)
5–12	901 (42.4)	398 (41.8)	503 (42.9)
13–15	222 (10.4)	95 (9.9)	127 (10.8)
16–18	81 (3.8)	40 (4.2)	41 (3.5)
19–28	222 (10.4)	102 (10.7)	120 (10.2)
29–38	262 (12.3)	119 (12.5)	143 (12.2)
39–48	239 (11.2)	102 (10.7)	137 (11.6)
49–58	128 (6.0)	67 (7.0)	61 (5.2)
> 59	67 (3.1)	28 (2.9)	39 (3.3)
Body mass index, n (%)	2,122 (100.0)	951 (44.8)	1,171 (55.2)
Under/Normal weight	979 (46.1)	400 (42.0)	579 (49.4)
Pre-obesity	665 (31.3)	340 (35.7)	325 (27.7)
Obesity	478 (22.5)	211 (22.1)	267 (22.8)
Skin color*, n (%)	1,611 (100.0)	751	820
White	724 (44.9)	360 (47.9)	364 (42.3)
Black	592 (36.7)	251 (33.4)	341 (39.6)
Others (brown, Asian, and indigenous)	295 (18.3)	140 (18.6)	115 (18.0)
Missing data	511 (24.0)	200 (21.0%)	311 (26.6)
Family monthly income	1,596 (100.0)	720	876
Low income	846 (53.2)	399 (55.4)	450 (38.4)
High income	747 (46.8)	321 (44.6)	426 (48.6)
Missing data	526 (24.8)	231 (24.3)	295 (25.2)
Residence area, n (%)	2,122 (100.0)	951	1,171
Urban	1,304 (61.5)	634 (66.8)	670 (57.3)
Missing data	5 (0.2)	3 (0.3)	2 (0.2)
Physical activity, n (%)	1,599 (100.0)	729	870
Inactive	1,078 (67.4)	440 (60.3)	638 (73.3)
Missing	523 (24.9)	222 (23.3)	301 (25.7)

*Self-declared.

### Phase Angle Centile Estimation

**Women:** The final age-related model for centile estimation was adjusted using the Box-Cox Cole and Green orig family distribution (BCCGo); there was no age transformation; mu = 10, sigma = 4, and nu = 4, and these were all associated with age.

**Men:** The final age-related model for centile estimation was adjusted using the Box-Cox Power Exponential orig family distribution (BCPE); there was no age transformation; mu = 13, sigma = 4, nu = 4, tau = 2, and these were all associated with age.

**FIGURE 1:**
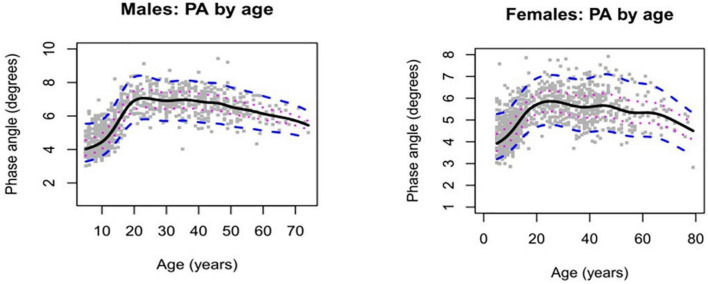
Phase angle by age in healthy males’ and female’s individuals. Solid lines represent median estimates together with 25th and 75th centiles (dotted lines) and 5th and 95th centiles (dashed lines).

The estimated phase angle percentiles showed for both sexes that the values increase through childhood, stabilize during most of adulthood, and decrease through late adulthood (Figure 1). The estimated percentiles and z-scores are shown in Table 2 for men and in Table 3 for women.

**TABLE 2:** Men’s phase angle percentiles and z-score.

Age	P5	P25	P50	P75	P95	−2	−1	0	+ 1	+2
5	3.28	3.63	4.03	4.56	5.54	3.18	3.49	4.03	4.86	5.99
6	3.33	3.69	4.09	4.61	5.54	3.23	3.55	4.09	4.90	5.96
7	3.38	3.75	4.15	4.67	5.56	3.28	3.61	4.15	4.95	5.95
8	3.44	3.83	4.23	4.75	5.6	3.33	3.68	4.23	5.02	5.96
9	3.52	3.92	4.33	4.84	5.67	3.41	3.77	4.33	5.10	6.00
10	3.62	4.03	4.45	4.96	5.77	3.5	3.87	4.45	5.22	6.09
11	3.74	4.18	4.61	5.12	5.91	3.62	4.01	4.61	5.38	6.22
12	3.91	4.37	4.81	5.32	6.11	3.77	4.19	4.81	5.58	6.42
13	4.11	4.60	5.05	5.58	6.37	3.96	4.41	5.05	5.84	6.67
14	4.34	4.86	5.34	5.88	6.68	4.18	4.67	5.34	6.14	6.97
15	4.59	5.15	5.65	6.2	7.01	4.42	4.94	5.65	6.47	7.31
16	4.86	5.45	5.97	6.54	7.35	4.67	5.23	5.97	6.81	7.65
17	5.11	5.74	6.28	6.86	7.69	4.92	5.51	6.28	7.14	7.99
18	5.35	6.00	6.55	7.14	7.97	5.14	5.77	6.55	7.42	8.28
19	5.53	6.21	6.77	7.36	8.19	5.31	5.97	6.77	7.64	8.49
20	5.66	6.35	6.91	7.5	8.32	5.43	6.11	6.91	7.78	8.62
21	5.74	6.45	7,00	7.59	8.39	5.51	6.20	7,00	7.86	8.68
22	5.79	6.5	7.05	7.62	8.41	5.55	6.25	7.05	7.89	8.69
23	5.81	6.52	7.06	7.62	8.39	5.56	6.27	7.06	7.88	8.67
24	5.8	6.52	7.05	7.6	8.35	5.56	6.27	7.05	7.86	8.62
25	5.79	6.50	7.03	7.56	8.29	5.54	6.26	7.03	7.81	8.56
26	5.77	6.48	7.00	7.52	8.23	5.52	6.24	7.00	7.76	8.49
27	5.74	6.46	6.96	7.47	8.17	5.49	6.21	6.96	7.71	8.42
28	5.72	6.43	6.94	7.43	8.12	5.47	6.19	6.94	7.67	8.37
29	5.71	6.42	6.92	7.41	8.09	5.45	6.18	6.92	7.64	8.33
30	5.7	6.42	6.91	7.39	8.07	5.44	6.18	6.91	7.62	8.31
31	5.7	6.42	6.91	7.39	8.07	5.43	6.18	6.91	7.62	8.31
32	5.71	6.44	6.93	7.40	8.07	5.43	6.20	6.93	7.63	8.31
33	5.72	6.45	6.94	7.42	8.09	5.44	6.21	6.94	7.64	8.33
34	5.72	6.47	6.96	7.43	8.11	5.44	6.22	6.96	7.66	8.35
35	5.73	6.48	6.97	7.44	8.12	5.44	6.23	6.97	7.67	8.37
36	5.72	6.48	6.97	7.44	8.13	5.43	6.23	6.97	7.67	8.38
37	5.71	6.47	6.96	7.43	8.12	5.41	6.22	6.96	7.66	8.37
38	5.68	6.45	6.93	7.4	8.10	5.39	6.2	6.93	7.63	8.35
39	5.66	6.42	6.90	7.37	8.07	5.36	6.17	6.90	7.60	8.33
40	5.63	6.39	6.87	7.34	8.04	5.33	6.15	6.87	7.57	8.30
41	5.61	6.37	6.85	7.31	8.02	5.30	6.12	6.85	7.54	8.28
42	5.59	6.35	6.82	7.29	8,00	5.28	6.10	6.82	7.52	8.26
43	5.57	6.33	6.8	7.27	7.98	5.27	6.09	6.8	7.5	8.26
44	5.56	6.32	6.79	7.26	7.98	5.26	6.08	6.79	7.49	8.26
45	5.56	6.32	6.78	7.24	7.97	5.25	6.07	6.78	7.48	8.25
46	5.54	6.3	6.76	7.22	7.95	5.24	6.06	6.76	7.46	8.24
47	5.52	6.27	6.72	7.18	7.91	5.22	6.03	6.72	7.41	8.20
48	5.48	6.22	6.67	7.12	7.85	5.18	5.98	6.67	7.35	8.14
49	5.43	6.16	6.61	7.05	7.78	5.14	5.93	6.61	7.28	8.07
50	5.39	6.11	6.55	6.98	7.71	5.10	5.88	6.55	7.21	8,00
51	5.35	6.07	6.5	6.93	7.65	5.06	5.84	6.5	7.16	7.95
52	5.33	6.04	6.46	6.88	7.60	5.04	5.81	6.46	7.11	7.90
53	5.31	6.01	6.42	6.84	7.56	5.02	5.79	6.42	7.07	7.86
54	5.29	5.98	6.39	6.8	7.52	5,00	5.76	6.39	7.03	7.82
55	5.27	5.96	6.36	6.76	7.48	4.98	5.74	6.36	6.99	7.78
56	5.25	5.93	6.32	6.72	7.43	4.96	5.71	6.32	6.94	7.73
57	5.22	5.9	6.28	6.67	7.38	4.94	5.68	6.28	6.89	7.68
58	5.19	5.86	6.24	6.62	7.32	4.91	5.65	6.24	6.83	7.62
59	5.16	5.82	6.19	6.56	7.26	4.88	5.61	6.19	6.77	7.55
60	5.13	5.77	6.14	6.51	7.19	4.85	5.57	6.14	6.72	7.49
61	5.1	5.74	6.09	6.45	7.13	4.83	5.54	6.09	6.66	7.43
62	5.07	5.71	6.05	6.41	7.08	4.81	5.51	6.05	6.61	7.38
63	5.05	5.68	6.02	6.36	7.03	4.79	5.48	6.02	6.57	7.33
64	5.03	5.65	5.98	6.32	6.98	4.77	5.46	5.98	6.52	7.28
65	5.01	5.62	5.95	6.28	6.94	4.76	5.43	5.95	6.48	7.23
66	4.99	5.59	5.91	6.24	6.88	4.74	5.41	5.91	6.43	7.18
67	4.97	5.56	5.87	6.19	6.83	4.71	5.38	5.87	6.38	7.12
68	4.94	5.52	5.82	6.13	6.77	4.69	5.34	5.82	6.32	7.06
69	4.9	5.47	5.77	6.08	6.70	4.66	5.30	5.77	6.26	6.99
70	4.86	5.43	5.72	6.01	6.63	4.62	5.26	5.72	6.19	6.92
71	4.82	5.37	5.65	5.94	6.55	4.58	5.20	5.65	6.12	6.84
72	4.77	5.31	5.58	5.86	6.46	4.53	5.15	5.58	6.04	6.74
73	4.71	5.24	5.51	5.78	6.37	4.48	5.08	5.51	5.95	6.65
74	4.65	5.17	5.43	5.69	6.27	4.43	5.01	5.43	5.86	6.54
75	4.59	5.10	5.35	5.60	6.17	4.37	4.95	5.35	5.76	6.44
76	4.53	5.03	5.27	5.52	6.07	4.31	4.88	5.27	5.67	6.34
77	4.47	4.96	5.19	5.43	5.97	4.26	4.81	5.19	5.59	6.24
78	4.42	4.89	5.11	5.35	5.88	4.21	4.75	5.11	5.50	6.14
79	4.36	4.82	5.04	5.27	5.79	4.15	4.68	5.04	5.41	6.05
80	4.30	4.75	4.97	5.18	5.70	4.10	4.62	4.97	5.33	5.95

**TABLE 3:** Women’s phase angle means estimates and z-score.

Age	P5	P25	P50	P75	P95	–2	–1	0	+1	+2
5	3.2	3.58	3.92	4.36	5.28	3.08	3.44	3.92	4.62	5.76
6	3.26	3.65	3.99	4.43	5.29	3.14	3.5	3.99	4.68	5.72
7	3.32	3.72	4.07	4.5	5.33	3.19	3.58	4.07	4.75	5.72
8	3.39	3.81	4.17	4.59	5.39	3.26	3.66	4.17	4.83	5.75
9	3.48	3.92	4.28	4.7	5.47	3.35	3.76	4.28	4.94	5.81
10	3.58	4.03	4.4	4.83	5.58	3.44	3.87	4.4	5.06	5.9
11	3.7	4.16	4.54	4.97	5.71	3.55	4.00	4.54	5.2	6.02
12	3.82	4.31	4.7	5.13	5.86	3.67	4.14	4.7	5.36	6.16
13	3.96	4.46	4.86	5.3	6.03	3.79	4.29	4.86	5.54	6.33
14	4.1	4.62	5.03	5.48	6.21	3.93	4.44	5.03	5.71	6.5
15	4.23	4.78	5.19	5.65	6.38	4.05	4.59	5.19	5.88	6.67
16	4.36	4.91	5.34	5.80	6.53	4.17	4.72	5.34	6.04	6.82
17	4.46	5.03	5.46	5.93	6.66	4.26	4.83	5.46	6.16	6.94
18	4.54	5.12	5.56	6.03	6.76	4.34	4.92	5.56	6.27	7.05
19	4.61	5.2	5.65	6.12	6.85	4.41	5.00	5.65	6.35	7.13
20	4.67	5.27	5.71	6.19	6.91	4.46	5.06	5.71	6.42	7.19
21	4.71	5.32	5.77	6.24	6.97	4.50	5.11	5.77	6.48	7.25
22	4.75	5.36	5.81	6.28	7.01	4.53	5.15	5.81	6.52	7.29
23	4.77	5.38	5.84	6.31	7.04	4.56	5.17	5.84	6.55	7.32
24	4.78	5.4	5.85	6.33	7.06	4.57	5.19	5.85	6.57	7.34
25	4.78	5.4	5.86	6.34	7.07	4.57	5.19	5.86	6.58	7.35
26	4.78	5.4	5.86	6.34	7.07	4.56	5.19	5.86	6.58	7.35
27	4.76	5.39	5.85	6.33	7.06	4.55	5.17	5.85	6.57	7.34
28	4.74	5.37	5.83	6.31	7.04	4.52	5.15	5.83	6.55	7.32
29	4.72	5.34	5.81	6.29	7.02	4.50	5.13	5.81	6.53	7.30
30	4.68	5.31	5.78	6.26	7.00	4.46	5.10	5.78	6.5	7.28
31	4.65	5.28	5.74	6.23	6.97	4.43	5.06	5.74	6.47	7.25
32	4.61	5.24	5.71	6.20	6.94	4.39	5.03	5.71	6.44	7.22
33	4.57	5.21	5.68	6.17	6.91	4.35	4.99	5.68	6.41	7.19
34	4.54	5.18	5.65	6.14	6.89	4.32	4.96	5.65	6.39	7.17
35	4.51	5.15	5.62	6.12	6.87	4.29	4.93	5.62	6.37	7.16
36	4.49	5.13	5.61	6.10	6.87	4.26	4.91	5.61	6.35	7.16
37	4.47	5.12	5.59	6.10	6.87	4.25	4.89	5.59	6.35	7.16
38	4.46	5.11	5.59	6.10	6.88	4.24	4.89	5.59	6.35	7.18
39	4.46	5.11	5.59	6.11	6.9	4.23	4.88	5.59	6.37	7.2
40	4.46	5.11	5.6	6.12	6.93	4.24	4.89	5.6	6.39	7.24
41	4.47	5.12	5.62	6.14	6.96	4.24	4.9	5.62	6.41	7.28
42	4.48	5.13	5.63	6.17	7.00	4.25	4.91	5.63	6.44	7.32
43	4.49	5.15	5.65	6.19	7.04	4.26	4.92	5.65	6.46	7.37
44	4.49	5.16	5.66	6.21	7.07	4.27	4.92	5.66	6.49	7.41
45	4.5	5.16	5.67	6.22	7.09	4.28	4.93	5.67	6.50	7.44
46	4.49	5.15	5.66	6.22	7.10	4.27	4.92	5.66	6.50	7.46
47	4.48	5.13	5.64	6.2	7.1	4.26	4.91	5.64	6.49	7.45
48	4.46	5.11	5.62	6.17	7.07	4.24	4.88	5.62	6.46	7.44
49	4.43	5.08	5.58	6.14	7.04	4.21	4.85	5.58	6.43	7.40
50	4.4	5.04	5.54	6.10	7.00	4.19	4.81	5.54	6.38	7.36
51	4.36	5.00	5.50	6.05	6.95	4.15	4.78	5.50	6.34	7.31
52	4.33	4.96	5.46	6.00	6.90	4.12	4.74	5.46	6.29	7.26
53	4.3	4.93	5.42	5.96	6.85	4.09	4.71	5.42	6.24	7.21
54	4.28	4.90	5.38	5.92	6.81	4.07	4.68	5.38	6.21	7.16
55	4.26	4.87	5.36	5.90	6.77	4.05	4.66	5.36	6.17	7.12
56	4.24	4.86	5.34	5.87	6.74	4.04	4.64	5.34	6.15	7.09
57	4.24	4.85	5.33	5.86	6.72	4.03	4.63	5.33	6.13	7.06
58	4.23	4.85	5.33	5.85	6.70	4.03	4.63	5.33	6.13	7.04
59	4.23	4.85	5.33	5.85	6.69	4.03	4.64	5.33	6.12	7.02
60	4.24	4.86	5.33	5.85	6.67	4.03	4.64	5.33	6.12	7.00
61	4.24	4.86	5.34	5.85	6.66	4.02	4.64	5.34	6.11	6.97
62	4.23	4.86	5.34	5.84	6.64	4.02	4.64	5.34	6.10	6.94
63	4.22	4.85	5.33	5.83	6.60	4.00	4.64	5.33	6.08	6.90
64	4.21	4.84	5.31	5.81	6.56	3.99	4.62	5.31	6.06	6.85
65	4.18	4.82	5.29	5.78	6.52	3.96	4.60	5.29	6.02	6.79
66	4.15	4.8	5.26	5.74	6.45	3.92	4.58	5.26	5.98	6.72
67	4.11	4.76	5.22	5.69	6.39	3.88	4.54	5.22	5.92	6.64
68	4.07	4.72	5.18	5.64	6.31	3.83	4.50	5.18	5.86	6.55
69	4.02	4.68	5.13	5.58	6.23	3.78	4.46	5.13	5.80	6.46
70	3.96	4.62	5.07	5.51	6.14	3.72	4.41	5.07	5.72	6.36
71	3.91	4.57	5.01	5.44	6.05	3.65	4.35	5.01	5.65	6.26
72	3.84	4.51	4.95	5.37	5.95	3.58	4.29	4.95	5.57	6.16
73	3.78	4.46	4.89	5.30	5.86	3.51	4.24	4.89	5.49	6.06
74	3.71	4.4	4.83	5.23	5.77	3.44	4.18	4.83	5.41	5.96
75	3.64	4.34	4.76	5.16	5.68	3.36	4.12	4.76	5.34	5.86
76	3.57	4.28	4.70	5.08	5.58	3.28	4.06	4.70	5.26	5.76
77	3.5	4.22	4.63	5.01	5.49	3.19	3.99	4.63	5.18	5.66
78	3.43	4.15	4.57	4.93	5.4	3.11	3.93	4.57	5.10	5.56
79	3.36	4.09	4.50	4.86	5.31	3.03	3.87	4.5	5.02	5.46
80	3.29	4.03	4.44	4.78	5.22	2.95	3.81	4.44	4.94	5.36

### Phase Angle Determinants

**Female:** In the final multivariable model, the relationship between phase angle and age was associated with BMI and family income (with a significant interaction) (Supplementary Table 1).

**Male:** In the final multivariable model, the relationship between phase angle and age was associated with BMI and skin color (with a significant interaction) (Supplementary Table 1).

## Discussion

This study used a large sample of healthy Brazilian individuals aged five to 80 years old to estimate phase angle, as measured by BIA. The models used allowed us to estimate smooth percentile curves as well as z-scores stratified by age and sex.

The estimated phase angle percentiles showed that the values increase through childhood, stabilize during most of adulthood, and decrease through late adulthood, which is consistent with the previously published meta-analysis (7). These findings reflect the physiological changes that occur throughout life. Considering that phase angle is an indicator of cell function and health, these findings reflect the different intracellular and functionality mechanisms involved in the cell membrane, which improve up to adult age but deteriorate during the late stages of life (7, 23).

We also found different phase angle determinants depending on sex. In both sexes, phase angle is associated with age and BMI. Previously study also showed that phase angle changes with sex and age and is associated with BMI (24). Its dependence on body composition is complex, being determined by BMI, %FM, and their interaction. However, in women there is an additional association with family income, with an age interaction; while in men there is an additional association with skin color, also with an age interaction. The comparison with the reference values already available was limited, since the statistical models, age groups and determinants were not differentiated between the studies (7, 24).

The causes of the differences observed between the sexes may include health status, cultural patterns, and biological and hormonal differences between men and women. As national economies progress, individuals with increased socioeconomic status may start taking a greater interest in their health, whereas people with a lower socioeconomic status may continue to struggle for calories. In this sense, the differences found between the determinants of phase angle in the different sexes may reflect the health inequalities still found between sexes and races (25–27).

Previous studies have shown an association between the level of physical activity and phase angle; however, we did not find this association (8). One possible explanation for our different results could be that most studies have included individuals diagnosed with a disease and they have not presented phase angle percentiles adjusted for sex and age.

This study is not free of limitations. A temporal relationship could not be defined due to the study’s cross-sectional design. This article included a convenience sample of participants from only one (South) of the five regions of Brazil. However, when the study data are compared with the latest national health surveys (28, 29), we observe that these are similar in the distribution of sex, skin color, BMI, and level of physical activity. Our study included a greater number of younger and fewer elderly individuals than the percentages in these age groups of the Brazilian population. The primary justification for including more young participants is that the data presented in this paper are part of an umbrella project with other objectives. There is a scarcity of studies that present phase angle values that include the elderly. One of the main limitations of including participants in this age group is that most have a chronic disease diagnosis. Despite the differences in the distribution of age groups, the uncertainties related to these data were expressed in confidence intervals.

To the best of our knowledge, it is the first attempt to apply the GAMLSS technique to predict future PA distributions using a healthy population and to cover most of the life cycle. In general, GAMLSS offers a flexible approach due to a large number of implemented distribution families. With GAMLSS, it is possible to assess the effect of specific parameters on the outcome variable distribution. The WHO has adopted the GAMLSS methodology for creating reference growth curves (30). The reference values in this study can be used more comprehensively in clinical practice for populations with mixed SES.

This study estimated a useful table of phase angle percentiles stratified by sex and age. To the best of our knowledge, it is the first attempt to apply the GAMLSS technique to estimate phase angle percentiles in a healthy population, covering most of the life cycle. We also showed that there are different phase angle determinants according to sex.

## Data Availability Statement

The raw data supporting the conclusions of this article will be made available by the authors, without undue reservation.

## Ethics Statement

The studies involving human participants were reviewed and approved by Comitê de Ética em Pesquisas da PUCRS. Written informed consent to participate in this study was provided by the participants’ legal guardian/next of kin.

## Author Contributions

RM: conception and design of the work, data collection, data analysis, interpretation, drafting the article, critical revision of the article, and final approval of the version to be published. PZ: conception and design of the work, data analysis, and interpretation, drafting the article, critical revision of the article, and final approval of the version to be published. EM: data collection, critical revision of the article, and final approval of the version to be published. All authors contributed to the article and approved the submitted version.

## Conflict of Interest

The authors declare that the research was conducted in the absence of any commercial or financial relationships that could be construed as a potential conflict of interest.

## Publisher’s Note

All claims expressed in this article are solely those of the authors and do not necessarily represent those of their affiliated organizations, or those of the publisher, the editors and the reviewers. Any product that may be evaluated in this article, or claim that may be made by its manufacturer, is not guaranteed or endorsed by the publisher.
